# Factors Associated With Low Birth Weight Among the Tribal Population in India: A Narrative Review

**DOI:** 10.7759/cureus.53478

**Published:** 2024-02-02

**Authors:** Arpana K Bhagat, Ashok M Mehendale, Komal N Muneshwar

**Affiliations:** 1 School of Epidemiology and Public Health, Datta Meghe Institute of Higher Education and Research, Wardha, IND; 2 Preventive Medicine, Department of Community Medicine, Datta Meghe Institute of Higher Education and Research, Wardha, IND

**Keywords:** low birth weight, prenatal risks factors, causes, risks factors, premature birth, mortality rate, neonates, lbws

## Abstract

Low birth weight (LBW) is defined by the WHO as a birth weighing less than 2500 g (5.5 lb). The highest burden of any nation is LBW infants. In countries where the frequency of neonate babies is high, short gestation is a major cause. LBW babies have an 11-13 times greater risk of delayed developmental milestones and other medical diagnoses. Greater than the global incidence, LBW prevalence is a severe public health problem in India. A comprehensive literature search was conducted using internet sources like PubMed, Web of Science, Cochrane Library, and Google Scholar. The words "birth weight," "abnormal birth weight," "LBWs," "neonates," "premature birth," "risks factors," "causes," "factors," "prevalence," and "frequency" were searched. In this review, we examine the causes of LBW, implementation of pre-birth prevention strategies, and post-birth multifaceted health promotion interventions. The mother's knowledge, dietary requirements, and prenatal services need to be addressed to decrease the prevalence of LBWs among tribal districts of India.

## Introduction and background

Low birth weight (LBW) is a significant problem in developing countries, especially in India. The epidemiological results show a significant association between newborn weight and prenatal and neonatal morbidity, with infants weighing less than 2500 g having mortality rates of almost 20 times greater than large infants [1]. Each year, 20.5 million live births are recorded in the world. Worldwide, 7.5 million newborn (27%) infants were LBWs, which accelerates the death rate in the infancy life period [2]. India is a country that accounts for over 40% of the world's burden, giving birth to under 8 million newborns per year. Premature and LBWs have an 11-13 times greater risk of having a poor newborn outcome than newborns of normal weight [3].

Almost 60% of the 7.5 million LBW newborns are born at term with delayed fetal growth [4]. Premature infants contribute to the surviving 40% of the total number of births [5]. The single factor with a significant influence on a newborn's chances of living, good growth, and development is weight at birth. LBWs generally fit into one of two groups: (a) premature labor (short gestation) or (b) infants with fetal development impairment [6]. The newborn's birth weight is the first measure taken followed by birth, in the initial hours, before there has been a major postnatal weight loss [7]. The newborn's first 28 days of life are the most dangerous in its life. Focusing on newborns is essential because the term "neonatal" indicates severe "endogenous variables" (such as LBW and delivery traumas) that affect an infant's life [8].

In nations with low populations, short gestation is the main factor leading to LBW newborns. In nations where the number of deaths is high, fetal developmental delay is the primary cause of cases, which can be seen in developing countries like India [9]. A preterm birth refers to a birth that comes initiated at 37 weeks of gestation, causing an estimated 15 million births; over 1 million preterm children die. Currently, the second-leading cause of fatality is prematurity. In 2015, the nation that had the greatest number of preterm deliveries was India [10]. In India, hemorrhage (38%), sepsis (11%), anemia (19%), abortion (7%), hypertension (5%), obstructed labor (5%), and other conditions (38%) are the major risk factors for LBW. Neonatal fatalities made up a larger section of under-five fatalities [11]. LBWs are strongly associated with a baby's chances of survival, growth, long-term health, and psychosocially development. A total of 95.6% of the 20 million estimated newborns who took birth each year with LBW are born in a poor state [12].

Nationwide, India is the place of birth for nearly 40% of LBW infants. With an estimated 12.8 million infants born in India alone, the prevalence of small for gestational age births was 47% [13]. The tribal groups are in various stages of development and contain various ethnic groups. The tribal population has a significant influence on infant and under-five mortality rates [14]. Madhya Pradesh, Maharashtra, Orissa, Gujrat, Rajasthan, Jharkhand, Chhattisgarh, Andhra Pradesh, West Bengal, and Karnataka include people with greater numbers of scheduled tribes [15]. Preterm birth and LBW infants are at high risk (48.1%), as are infants with birth asphyxia and trauma (12.9%), newborn pneumonia (12%), sepsis (5.4%), and birth defects (4%). Two of the main factors that cause newborn deaths in India are diarrhea (3.1%) and injuries (0.9%) [16]. India has a higher weightage of LBWs than the majority of the nations, which is an important public health issue [17]. Even though the incidence of premature LBW has decreased considerably over the last 10 years, from 20.4% to 16.4%, it continues to be India's major public health problem [18]. The WHO defines an LBW baby as a baby who weighs below 2500 g at birth [19].

Preterm, term, and post-term are the three groups that can be used to classify LBW according to gestational age. Preterm babies include newborns born before 37 weeks of pregnancy (below 259 days); term babies include babies born between 259 and 293 days of gestational period. Babies born after a gestational period of 294 days or after 42 complete weeks may be post-term. Preterm babies may be classified into two groups according to the gestation period: extremely preterm (born before 28 weeks) and extremely premature (32 weeks) [9]. Causes and risk factors of premature and LBW are shown in Table 1.

**Table 1:** Causes and risk factors of premature and low birth weight

S. No.	Causes	Risks factors	Examples
1.	Spontaneous preterm birth [20]	Age at pregnancy and pregnancy spacing	Adolescent pregnancy, advanced maternal age, or short inter-pregnancy interval
		Multiple pregnancies	Increased rates of twin and higher-order pregnancies with assisted reproduction
		Infection	Urinary tract infections, malaria, HIV, syphilis, bacterial vaginosis
		Underlying maternal chronic medical conditions	Diabetes, hypertension, anemia, asthma, thyroid disease
		Nutritional	Undernutrition, obesity, micronutrient deficiencies
		Lifestyle/work-related	Smoking, excess alcohol consumption, recreational drug use, excess physical work/ activity
		Maternal psychological health	Depression, violence against women
		Genetic and other	Genetic risk, e.g., family history and cervical incompetence
2.	Provider-initiated preterm birth [21]	Medical induction or cesarean birth for obstetric indication or fetal indication	The risk factors for preterm delivery indicated by a provider overlap with those for preterm birth that occurs naturally

## Review

Methodology

**Figure 1:**
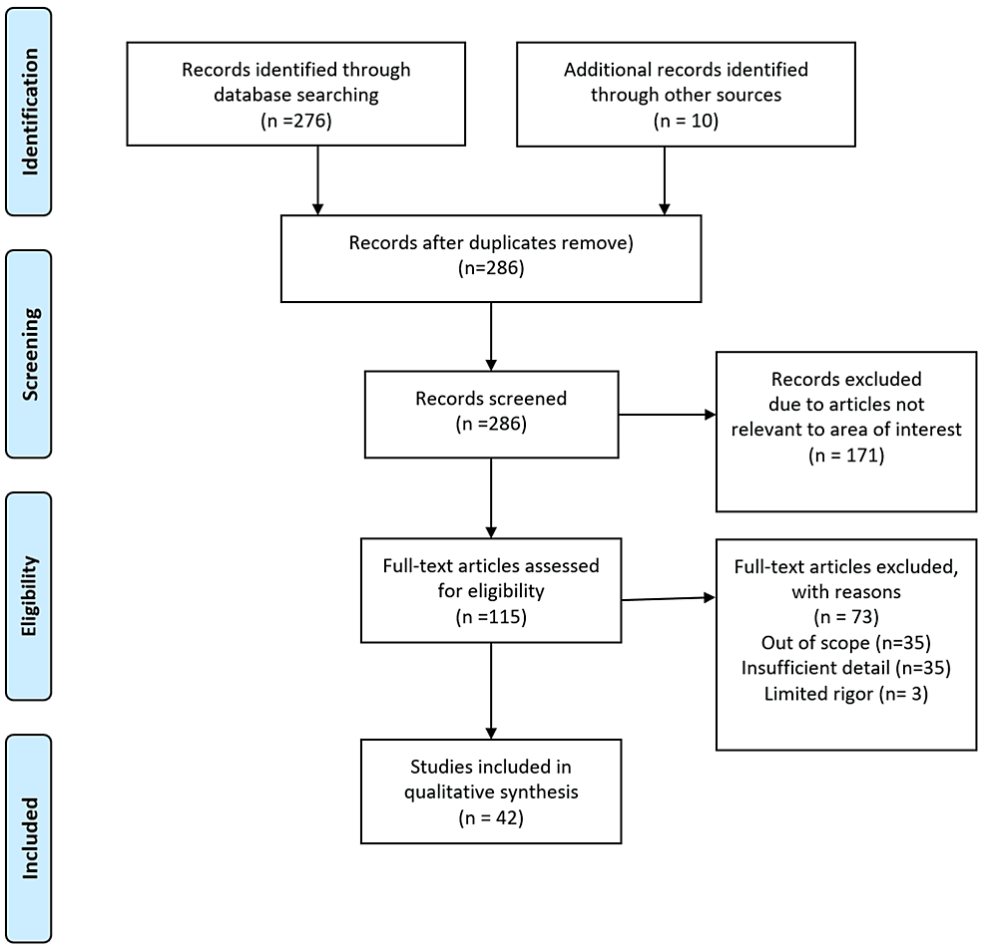
Inclusion and exclusion criteria for this study

A comprehensive literature search was conducted using internet sources like Google Scholar, PubMed, Web of Science, and Cochrane Library. The search strategy was used to look for relevant studies using certain terms. The keywords were "birth weight," "abnormal birth weight," "LBWs" "neonates," “mortality rate,” "premature birth," "risks factors," "causes," "factors," "maternal risk factors," "prevalence," "frequency," and "LBW." Additionally, MeSH terms like "newborn OR neonate OR infant AND birth weight OR LBWs OR abnormal birth weight OR underweight AND risk factors OR causes AND prevalence" and other synonyms were also utilized to advance PubMed search. The Preferred Reporting Items for Systematic Reviews and Meta-Analyses (PRISMA) flow diagram is given below in Figure 1.

Discussion

LBWs are defined as newborns weighing lower than 2500 g who are induced generally by intrauterine growth restriction (IUGR), preterm birth, or both. Birth weight has a positive association with a newborn's chances of survival, development, lifelong health problems, and growth psychosocially [22]. The gestational age may be made as a predictor of delayed immunization if preterm labor is the primary cause of LBW [23]. In this study, maternal anemia and hypertension were substantially correlated with LBW. Other studies have come up with similar findings. Anemia could impair the fetus's capacity to obtain enough oxygen, which would hinder healthy intrauterine growth. The availability of nutrients is thought to be significantly caused by the mother's pressure and this change decreased placental blood flow and resulted in LBW [24]. The present research study found links between LBW and various maternal and socioeconomic factors. This study determined that teenage women have a significantly greater chance of giving birth to LBW babies [24]. In India, as part of the government's healthcare mission, Accredited Social Health Activists (ASHAs) and Anganwadi workers are responsible for measuring the height and weight of babies delivered in the family on the day of the delivery [12]. The WHO recommends a minimum of four appropriate prenatal and postnatal visits, during which the mother's weight, height, urine, and blood values are measured, she receives a tetanus shot, and additional dietary supplements are prescribed [18]. In high-priority districts, encouraging community-based education on better mother and baby care as well as home-based treatment for neonate infections might significantly improve newborn survival. To secure the use of mother and baby healthcare services, effective consultations regarding particular maternal and child health services required should be given to pregnant women during their initial antenatal care visits [16]. In addition, there must be an early start to comprehensive education programs focused on women's self-care, overall well-being, and nutrition. If they want to secure a better future for newborns and their mothers, they could assist with reducing the burden of LBW in healthcare systems, as well as by reducing resulting mortality risks [25]. The Indian government offers a number of welfare programs for pregnant mothers that deal with concerns about food habits during pregnancy, early identification of issues, and socioeconomic strain/out-of-pocket costs related to antenatal care and delivery. Additionally, the Indian government is working on delivering prenatal and postnatal services, and dietary requirements [2]. A breastfed child is more likely to survive than one that is fed artificially. The LBW newborn is protected against certain illnesses and early malnutrition by prolonged breastfeeding [6].

Janani Suraksha Yojana (JSY) 

Janani Suraksha Yojana (JSY) is also known as the Safe Motherhood Intervention Scheme. JSY of the National Rural Health Mission (NRHM) encourages institute delivery among antenatal mothers whose socio-economic status is not well [26]. JSY, a comprehensive conditional cash transfer program developed to encourage low socioeconomic status women to give birth in health facilities, was introduced by the Indian government in April 2005 according to the broad NRHM guidelines. The program was then implemented in an effort that it would help reduce the maternal mortality rate (MMR) [27]. The initial National Maternity Benefit Scheme served as the model for the new program. For a price of Rs 500 for each pregnancy, the program provided women with low-income antenatal and postpartum services for their first two live births [28]. JSY offered cash benefits to a woman if the birth occurred at a public or established private healthcare facility in the target nations (Assam, Bihar, Chhattisgarh, Jammu, and Kashmir, Jharkhand, Madhya Pradesh, Orissa, Rajasthan, Uttarakhand, and Uttar Pradesh) [29].

Janani Shishu Suraksha Karyakram (JSSK)

The Indian government introduced JSSK on June 1st, 2011. This program is for pregnant women who deliver babies at policy facilities [30]. Under this program, sick infants (including those with birth-related medical and medical conditions) obtain free treatment at public health facilities, as well as free diagnostics, blood supplies, blood transfusions, free diets, and exclusion from all types of service fees for the first 30 days after delivery [31]. In recognition of the effectiveness of this secure pregnancy and motherhood program, an important program known as JSSK was initiated in June 2011 with the goal of reducing out-of-pocket costs among women who are expecting sick infants. The mother and her infant receive crucial care within 48 hours [32]. It is estimated that more than 10 million infants, from urban and rural areas, get into facilities each year, with benefits of the JSSK scheme [31].

Navjaat Shishu Suraksha Karyakram (NSSK)

The Ministry of Health and Family Welfare introduced "NSSK," a new program on basic neonate care and resuscitation, which aims to prevent hypothermia and infection and initiate breastfeeding or basic neonate resuscitation [33]. Neonate care and recovery are essential starting points of any newborn program required for the most effective establishment in life. With the new program, all deliveries will have healthcare providers who are educated on basic infant care and treatment. Using this strategy will raise infant survival rates and significantly reduce newborn fatality rates [34].

Home-Based Newborn Care (HBNC)

The government of India started the HBNC scheme in 2011. The objectives of this scheme are to improve community neonate care practices, earlier detection of newborn diseases, and appropriate referral through home visits [35]. These preventive care services for expecting mothers and babies will continue to be delivered by ASHAs (grassroots-level primary healthcare workers). For up to 40 days after the time of birth, ASHAs visit every newborn in accordance with the designated timetable [35]. They provide infant care through a series of home visits and skills, such as taking neonate temperature, ensuring warmth, promoting exclusive breastfeeding by educating the mother on proper positioning and attachment for starting and continuing breastfeeding, diagnosing and counseling in the event of a breast-feeding issue, encouraging hand wash, skin care, cord clean, and eye care, promoting health, and giving women and their family’s support on different issues [36]. They determine if a newborn is a high risk (preterm or LBWs) and use guidelines to care for such LBWs or preterm babies [36]. They increase the frequency of home visits to keep an eye on the weight gain, keep the baby warm, and do regular healthcare checks in case the mother and family need support and counseling. ASHAs educate mothers on exclusive breastfeeding, and if required, provide a newborn baby with a cup of milk and a spoon [37].

Kangaroo Mother Care (KMC)

Skin-to-skin contact with the importance of exclusive breastfeeding is often focused on in the Indian government's program on the care of newborns. The government-supported scheme, Facility Based Integrated Management of Neonatal and Childhood Illness (F-IMNCI), has been promoting KMC [38]. KMC can help in reducing the death rate of LBWs. "Kangaroo mother care," a less technology-based and cost-effective strategy, is described as "a conventional, practice care system for neonate/or LBWs based on skin-to-skin contact linked to mothers and care provider” [39]. Providing care to an infant requires warmth, a mother's milk, infection control, safety, and emotional support. Based on the World Health Organization, KMC is a facility that provides care for LBW and premature newborns. There are further indications that KMC increases the frequency, duration, and exclusivity of breastfeeding in India [39].

Indian Newborn Action Plan (INAP)

Launched in September 2014, the objective of INAP is to reduce the newborn fatality rate (per 1000 live births) by 2030 [40]. The scheme is to provide KMC, prenatal pre-conception care, care during labor, initial premature babies' appropriate neonate health care, treatment of small and in critical condition neonates, and care beyond neonate survival to 90% of mothers [40]. INAP focuses on knowledge, and fast growth is possible, especially when using the integrated strategy of INAP, linking significant interventions across the continued care, from prenatal services to postpartum periods, and focusing on natural connections linked to maternal and newborn health care [41]. From preventive care to after-delivery treatment, a broad planning program is provided. The six elements of the intervention indicated previously must be implemented to reduce neonatal mortality [42].

Interventions taken under the National Health Mission (NHM) on premature and LBW babies are shown in Table 2.

**Table 2:** Interventions taken under the National Health Mission (NHM) on premature and LBW babies LBW: low birth weight.

S. No.	Programs	Year	Objectives
1.	Janani Suraksha Yojana (JSY) [27]	2005	Safe motherhood preventive measures to condition cash transfer programs designed to encourage low-socioeconomic status women to give birth in health facilities.
2.	Janani Shishu Suraksha Karyakram (JSSK) [32]	2011	Zero out-of-pocket expenditure within 48 hours, and the woman and her newborn get critical treatment.
3.	Navjaat Shishu Suraksha Karyakram (NSSK) [33]	2009	Basic neonate care and resuscitation to address crucial interventions of care at birth, and training program.
4.	Home-Based Newborn Care (HBNC) [36]	2011	Provision of essential neonate care to all neonates, special care of premature or LBW babies, initial detection of diagnosis followed by referral, and promotion to the family for good healthy practices by ASHA workers.
5.	Indian Newborn Action Plan (INAP) [41]	2014	Linking important interventions across the continued care, from the antenatal to postnatal periods, focusing on natural connections between reproductive, prenatal, neonate, and child health care.

## Conclusions

Almost 20 million newborns worldwide are born underweight. Finding the causes of LBW and implementing preventative strategies are the main challenges in public health. This research tried to determine the prevalence of LBWs and the risk factors that affect them. To reduce the frequency of LBWs in India, it has been critical to address antenatal services and women's education. Also, certain programs have been initiated by the government. These interventions aid in the nation's achievement of the Millennium Development Goals by reducing not only the prevalence of LBWs but also the problems related to them. Continued implementation of multifaceted health promotion interventions is needed to address these factors effectively. The mother's education, dietary requirements, and antenatal services need to be addressed to decrease the prevalence of LBWs among the tribal states in districts of India. Prenatal care is key to services in the monitoring of the newborn. Priority to the regular monthly weights and growth monitors for newborns may be adopted as an effective public health program to decrease LBWs among the tribal-dominated states in India. Further study is necessary to improve this tool and evaluate its effectiveness and acceptance in populations with various preterm birth prevalence rates and various healthcare systems. Before it is implemented for use in public health settings, it is also necessary to assess the economic effectiveness in reducing the burden of mortality due to preterm birth.
